# Supplementary material for: Active site specificity profiling datasets of matrix metalloproteinases (MMPs) 1, 2, 3, 7, 8, 9, 12, 13 and 14
Source: Data Brief. 2016 Feb 22;7:299–310. doi: 10.1016/j.dib.2016.02.036 (PMC4777984; doi:10.1016/j.dib.2016.02.036)
Supplement: Supplementary file 10 — Supplementary material [file mmc10.zip › WebPICS_hMMP8_T_1%/P2prime.html]

 

PICS results


|  |  |
| --- | --- |
| **P2prime\_H**  12 in 335 sites   3.6 %    effects > 10 perc. pnts.  (vice-versa in brackets)  P3\_T: 19.3 (12.2)   P1prime\_C: 19.9 (14.0) |  |
| **P2prime\_I**  35 in 335 sites   10.4 %    effects > 10 perc. pnts.  (vice-versa in brackets)  P2\_D: 14.5 (18.2) |  |
| **P2prime\_L**  60 in 335 sites   17.9 %    effects > 10 perc. pnts.  (vice-versa in brackets)  P1prime\_I: 14.5 (16.7) |  |
| **P2prime\_M**  21 in 335 sites   6.3 %    effects > 10 perc. pnts.  (vice-versa in brackets)  P2\_M: 10.4 (16.8) |  |
| **P2prime\_Q**  20 in 335 sites   6.0 %    effects > 10 perc. pnts.  (vice-versa in brackets)  P2\_Y: 12.8 (10.7) |  |
| **P2prime\_T**  24 in 335 sites   7.2 %    effects > 10 perc. pnts.  (vice-versa in brackets)  P2\_W: 10.4 (35.7) |  |
| **P2prime\_W**  6 in 335 sites   1.8 %    effects > 10 perc. pnts.  (vice-versa in brackets)  P2\_F: 59.8 (15.6) |  |
